# The Complex Pre-Execution Stage of Auditory Cognitive Control: ERPs Evidence from Stroop Tasks

**DOI:** 10.1371/journal.pone.0137649

**Published:** 2015-09-14

**Authors:** Bo Yu, Xunda Wang, Lin Ma, Liang Li, Haifeng Li

**Affiliations:** 1 School of Computer Science and Technology, Harbin Institute of Technology, Harbin, 150001, China; 2 Software College, Harbin University of Science and Technology, Harbin, 150001, China; 3 Department of Psychology and Beijing Key Laboratory of Behavior and Mental Health, Speech and Hearing Research Center, Key Laboratory on Machine Perception (Ministry of Education), PKU-IDG/McGovern Institute for Brain Research, Peking University, Beijing, China; University of Rome, ITALY

## Abstract

Cognitive control has been extensively studied from Event-Related Potential (ERP) point of view in visual modality using Stroop paradigms. Little work has been done in auditory Stroop paradigms, and inconsistent conclusions have been reported, especially on the conflict detection stage of cognitive control. This study investigated the early ERP components in an auditory Stroop paradigm, during which participants were asked to identify the volume of spoken words and ignore the word meanings. A series of significant ERP components were revealed that distinguished incongruent and congruent trials: two declined negative polarity waves (the N1 and the N2) and three declined positive polarity wave (the P1, the P2 and the P3) over the fronto-central area for the incongruent trials. These early ERP components imply that both a perceptual stage and an identification stage exist in the auditory Stroop effect. A 3-stage cognitive control model was thus proposed for a more detailed description of the human cognitive control mechanism in the auditory Stroop tasks.

## Introduction

One of the most intriguing challenges in cognitive neuroscience is to explain the precise neural mechanisms that underlie cognitive control. A central question about the nature of cognitive control is the temporal profiles of the mechanism [1–4].

Preliminarily, the conflict monitoring theory proposes a conflict detection stage and a conflict resolution stage involving in cognitive control [5, 6]. This theory has partially reflected the temporal profiles of the cognitive control mechanism: the detection stage generates and transmits signals to specific brain areas that execute conflict resolution, while the resolution stage represents the execution of conflict resolution. However, it is not very clear whether the conflicts produce cognitive control effects in the sensory processing stage.

The Stroop effect is an important experiment paradigm in the study of cognitive control and conflict monitoring mechanism [7–16]. The Stroop effect, named after John Ridley Stroop, refers to the phenomenon when participants were presented with color-words and were required to name the colors, slower and less accurate responses were revealed for the incongruent trials than for the congruent ones [17]. The incongruent stimuli consist of words with different colors and semantic meaning, and the congruent stimuli consist of color-meaning corresponding words. Similar to the original Stroop effect, many extended paradigms have been proposed in previous studies, for instance, the auditory Stroop paradigm [18], the emotional Stroop paradigm [19], and the spatial Stroop paradigm [20]. In addition to the classical research fields such as attention, cognition and language, the Stroop effect has been employed in recent studies to various other fields like memory [21–23], addiction [9, 24, 25] and emotion [26–28]. The extensive usage of the Stroop paradigms indicating that the conflict control is a core function of brain executive system involving many aspects of human performance, and this function can be well characterize by studies using the Stroop paradigms.

Although a lot of evidence from many classical visual Stroop studies had proved the conflict detection-resolution mechanism corresponding to the conflict monitoring theory, no significant conflict control effects in the perceptual stage have been observed. Some previous functional magnetic resonance imaging (FMRI) or positron emission tomography (PET) studies on the visual Stroop paradigm had confirmed that the anterior cingulate cortex (ACC) [3, 29, 30], the medial and lateral prefrontal cortex (MPFC and LPFC) and the parietal lobe [31–33] involve in cognitive control and conflict monitoring. Kerns found that a later ACC conflict-related activity is a prediction of a subsequent increase in prefrontal cortex (PFC) activity and behavioral adjustments, which means that the ACC’s function in visual Stroop effect is conflict detection [2]. Such PET and FMRI studies have proved the existence of conflict detection-resolution mechanism. Meanwhile, the majority of ERP studies using the classical visual paradigm also show correspondence with conflict monitoring theory. Liotti [34], Markela-Lerenc [15] and West [35] had confirmed the conflict detection-resolution mechanism in their ERP studies. Furthermore, in another study, Larson and colleagues [36] employed a sequential analysis to observe the short-term memory effect on these components, and found that the late slow-wave component (representing the conflict resolution stage) indicated a significant sequential effect, whereas the 450 ms component (the N450, the source of which located at the ACC, representing the conflict detection stage) had no such effect. These results suggested that the late slow-wave component has a higher correlation with the conflict monitoring or conflict adaptation than the N450. Despite those conflict-related Stroop ERP components under different experimental conditions (e.g. early components peaked at about 100 ms and 200 ms, later components after 300 ms and the slow-wave component), the previous studies in the classical visual Stroop effect have casted light on the correlation of later conflict interference effects and conflict adjustment, while perceptual effects might have been ignored to some extent.

However, as what has been discussed for a long time, a complete cognitive control process should include perceptual processes (such as a sensory process and an identification process) before the decision making and responding [37]. Early ERP components always individually or synthetically reflect such perceptual processes. Auditory version studies, in contrast with classical visual version studies, showed that cognitive control can modulate more automatic processing stage [7, 16].

From 1975, due to the great difference between the auditory and the visual modality, the auditory Stroop effect began to attract people’s increasing attention in the cognitive control mechanism study. In an auditory Stroop task, participants are typically required to respond to the acoustic properties of speech stimuli, and ignore the word meanings [7, 38]. Comparing to a visual paradigm, an auditory paradigm excludes the effect from word shape.

Several previous auditory Stroop studies tried to characterize a similar cognitive control mechanism in the visual paradigms. Respectively, Hamers [39] and Cohen [40] performed the behavioral experiments for the auditory Stroop effect, and drew a consistent conclusion to the visual one: incongruent auditory stimuli led to prolonged reaction time and less accuracy rate. Afterwards, a series of behavioral auditory Stroop studies also supported this conclusion [7, 41–43]. Evidence from FMRI had also reported the same results. For instance, a FMRI research on both the visual and the auditory Stroop effects proved the existence of similar conflict-related brain areas in both paradigms (e.g. ACC, bilateral inferior frontal gyrus, anterior insula, and parietal lobe) [44]. However, it is obvious that these behavioral or low-temporal-resolution studies are not clear enough to confirm the precise temporal dynamics of the cognitive control mechanism.

Due to the higher temporal-resolution, ERP studies are expected to obtain more valuable and reliable results. Although several ERP studies have been done to the auditory Stroop tasks, the results revealed some noticeable inconsistencies, especially in the conflict detection stage [7, 16].

To our knowledge, only two ERP studies using auditory Stroop task had found both of the early stage and the later stage of cognitive control, thus could provide little support for the “supramodal” conflict detection-resolution mechanism. Donohue and Liotti [16] confirmed the conflict detection-resolution mechanism in their auditory study. They found an early ERP component peaked at 300 ms (called Ninc, 200 ms~500 ms) followed by a late-SP (late Sustained Positivity, from 500 ms to 800 ms), and proposed that these two components respectively corresponded to the N450 and the late-SP components in the former visual research [34]. The sequential analysis they employed also suggested that the later stage mainly involved in cognitive adaptation. Another earlier study conducted by Lew [45] provided evidence for the Stroop interference effect in both perceptual and post-perceptual (or response) processes.

Unfortunately, other auditory ERP studies did not observed the complete conflict detection-resolution mechanism. For instance, Buzzell and his colleagues [46] demonstrated that the Ninc is predictably modulated by individual differences in cognitive style and confirmed the Ninc reliably indexes auditory Stroop conflict by an auditory spatial Stroop task. Henkin and his colleagues [7] identified that the early N1 effect was modulated by cognitive control. However, no significant SP (Sustained Positivity) modulations were observed in their study.

A remarkable difference between the auditory and the visual study results is that the latencies of the Ninc or the N1 effects (which were regarded as the conflict detection stage) was different from the N450 found in the visual paradigm. The auditory model given by Donohue [16] suggested that the 150 ms shorter latency of the Ninc than the N450 possibly due to the inter-modality difference in cognitive control processing schedules. Additionally, it may also due to the additional processing delay in the secondary cortex in visual processing (secondary or association areas), or due to the simple two-choice nature of their experimental design [16]. Meanwhile, as the N1 modulation has been identified to represent the conflict detection in other previous auditory tasks [47, 48], it may also exist in the auditory Stroop tasks. Previous visual researches did not prove that the early modulations (the N1 effect and the Ninc) involved in cognitive control possibly because of the higher automatic processing of the color in the visual paradigm, or due to other paradigm limitations. Nonetheless, given such the N1 effects and the Ninc happened much earlier than the N450, auditory Stroop paradigm might be more applicable to measure all modulations related to the early stage of cognitive control and conflict monitoring.

Another debate is about the early stage of cognitive control itself—different ERP components were observed to represent the conflict detection stage in the previous ERP studies. Two studies [7, 45] reported that the N1 component involved in the conflict monitoring and cognitive control mechanism, thus gave support to the point that the N1 modulation generates the auditory modality-specific sensory signal (implemented the conflict detection stage) before the conflict resolution. Instead, other two studies [7, 16] identified Ninc as the index of conflict detection but not N1 effect. It seems that both the Ninc and the N1 effect were produced by auditory Stoop interference, but have not been identified simultaneously in one study.

Taken together, the conflict monitoring mechanism have not yet been well characterized in the previous studies. The specific processes and the temporal files of the auditory Stroop effect still require in-depth study and discussion. On one hand, exploring the interference effects of auditory Stroop task would help us in obtaining a better understand of the similarities and differences between the auditory and the visual modalities, especially in the pre-execution stage of cognitive control and conflict monitoring. On the other hand, it is necessary to perform further analysis on the time course of the early ERP components in the auditory Stroop tasks, in order to explore whether cognitive control can influence the early perceptual processes.

Another motivation of this study relates to the lingual factors in the Stroop effect. Besides the extensive studies in English, it is necessary to perform different language situations for the sake of comparing study. In the previous studies, Henkin *et al*. made an excellent supplement to the general Stroop studies in English by using Hebrew [7]. In comparison with these languages, the oriental languages like Chinese version of the auditory Stroop almost got no attention. The use of Chinese stimuli has just got its start and the related researches were quite insufficient [49]. Above all, studying those early perceptual ERP components in this oriental languages version of the auditory Stroop task compared with other languages one mote the full understanding of the cognitive control mechanism.

To confirm the existence of the conflict detection-resolution mechanism and the modulations of those early ERP components, the present study compared the present study with the previous auditory Stroop studies. We hoped to find modulations of all those corresponding early ERP components (the N1 effect and the Ninc) that relate to cognitive control, and draw the conclusion that our auditory Stroop effect is similar to the other language versions. Furthermore, we wished to identify that the pre-execution stage of the cognitive control mechanism contains two specific detection stages: a perceptual stage and an identification stage. Finally, by synthesizing the present study with the previous studies, a more complete model of conflict monitoring and cognitive control would be developed.

In previous studies, a common concern is about the physical differences between congruent and incongruent conditions that might potentially drive different modulations in the early sensory components. We carefully designed balanced the congruent and incongruent stimuli sets. Both sets contained the same physical attributes over the corresponding conditions: two words, and each word have two loudness levels, two genders. Therefore, after respectively averaging the congruent and the incongruent trials to get each ERPs and getting the difference of the two ERPs by substruction, the ERP effects produced by the bottom level properties would be completely eliminated. Therefore, any ERP modulation seen in the auditory components are just subject to the differences of the stimuli type (congruent vs. incongruent). Besides, because all our expected ERP effects should be located in the frontal and central areas, they are proposed to implement higher-order executive functions. In addition, one method was utilized for improving the measurement accuracy in this study: analyses of mean amplitude over smaller time intervals (in relative to the previous studies) in all periods after the stimulus onset to ensure every modulation of interest ERP component could be well measured.

## Method

### 2.1 Participants

21 healthy students from Harbin Institute of Technology (10 females and 11 males; age, 21~25 years, mean = 22.8) were invited to participate in this experiment. All of them were right-handed and none had a history of neurological disease. All had normal vision and normal hearing. All of the participants provide their written consent to participate in this study. The present study was approved by the applied ethics research center of the Harbin Institute of Technology. Each participant was told the procedure of the task before following the instructions to complete the experiment. After the experiment they were paid for their participation.

### 2.2 Stimuli and Task

Auditory Stimuli were recorded by two adult (male and female, for eliminating the gender difference) native Chinese speakers, all stimuli were mixed uniformly and were presented together. The congruent stimuli consisted of the word /Da/ (means loud voice) spoken loudly and the word /Xiao/ (means low voice) spoken lowly. The incongruent stimuli consisted of the word /Da/ spoken lowly and the word /Xiao/ spoken loudly. The loudness difference between the low stimuli and the loud stimuli were adjusted at 20dB (low stimuli were 20dB lower than the original recordings, which were used as loud stimuli). These two words have similar consonant structure and duration, and have similar appearance frequency in Chinese daily language. Table 1 presents the main stimuli characteristics. Auditory stimuli were played out through the AMD high definition audio device sound card and the HiVi h5 speaker. To avoid physical differences between two types of stimuli which might potentially influence the early sensory components, both the congruent and incongruent stimuli sets consisted of the same physical attributes (two words, and each word have two loudness levels, two speaker-genders), hence the experiment design had balanced the ERP effects produced by the bottom level properties of two kinds of stimuli.

**Table 1:** Stimulus List.

Stimuli (Word meaning)	Gender	Relative voice volume (dB)	Conditions
/Da/ (Loud voice)	male	0	Congruent
/Da/ (Loud voice)	male	-20	Incongruent
/Da/ (Loud voice)	female	0	Congruent
/Da/ (Loud voice)	female	-20	Incongruent
/Xiao/ (Low voice)	male	0	Incongruent
/Xiao/ (Low voice)	male	-20	Congruent
/Xiao/ (Low voice)	female	0	Incongruent
/Xiao/ (Low voice)	female	-20	Congruent

After electrode application, participants were seated in a comfortable chair in a quiet and dimly lit room, approximately 60 cm in front of a computer screen. During the task, the instructions were presented on the computer screen, which was auto-controlled by the Presentation 15.0. Keyboard was also placed in front of the participant (distance 30 cm). Only two buttons were used: the upper button (↑) and the lower button (↓).

Before the formal tasks, participants were asked to do some additional short tasks to get familiar with the voice volume. Participants were then given a rest, and afterwards were asked to click on the upper button (↑) to begin the task. During the task, participants were instructed to fixate their eyes on a “+” located on the centre of the computer screen and respond as quickly as possible when they heard the auditory stimuli.

In the task, participants were instructed to identify the volume of the stimuli and press the upper button (↑) for a loud volume or the lower button (↓) for a low volume, regardless of the word meanings.

The task consisted of 320 trials. Four kinds of stimuli were randomly presented with equal probability (0.25) by audio amplifier with a loudness limitation at 60 dB. The duration of every auditory stimulus was 400 ms, and the interval between every two stimuli was randomly adjusted to 2000, 2100, 2200, 2300 or 2400 ms.

### 2.3 Recording

Each participant’s brain electrical activity was continuously recorded from 64 sites on the scalp using Ag/AgCl electrodes mounted in an elastic cap (NeuroScan Inc., Herndon, VA, USA), referenced to the reference electrode located at the middle of Fz and Cz (had been re-referenced to the average of the left and right mastoids in the data processing). Amplifier settings were as the following: band pass filter (0.01–100 Hz), sampling rate (1000 Hz), electrode impedance (< 10 kΩ). Synchronous behavioral performance was recorded by the Presentation 15.0.

### 2.4 Data Analysis

#### 2.4.1 Offline EEG Data Analysis

Offline EEG data processing was performed by using the Scan 4.5 (NeuroScan Inc., Herndon, VA, USA). Firstly, manually excluded trails which containing large muscle artifacts or extreme voltage offsets (identified by visual inspection). We then removed the VEOG from the raw EEG signals using a regression method (implemented by means of an algorithm after the manual setting of ocular artifact reduction parameters: trigger threshold (10%), minimum number of sweeps (20), and duration (400 ms)). Then the EEG was segmented into the epochs from 200 ms pre-stimulus to 823 ms post-stimulus. After epoching, the automated artifact rejection procedure were performed to reject trials in which the voltage exceeded the normally defined criteria (70~150 μv). The automatic rejection rate was limited to 20% (relative to the remaining trials of the manual rejection). Baseline correction was then performed for the artifact-free data. In the following steps, the data were averaged separately for each stimuli condition (incongruent or congruent), then the averaged EEG data were re-referenced to the average of the left and right mastoids and digitally filtered with a 30 Hz low-pass filter. Finally, the grand average waveforms of all participants were computed for each trial type, as well as for the difference waves (incongruent minus congruent).

#### 2.4.2 Statistical Analysis of the ERP data

For the purpose of focusing on the early components and investigating the early ERP components more accurately, we applied a statistical analysis method similar to the previous studies [16, 34], but with smaller time windows and in broader post-stimulus periods. In some auditory cognitive control task, many studies consider Fz and Cz as the most frequently analyzed locations since maximal amplitude for the N1, the N2 and the MMN is achieved [50–53]. Therefore, 12 fronto-central electrodes (F1, Fz, F2, FC1, FCz, FC2, C1, Cz, C2, CP1, CPz, CP2) were included in the analysis. Additionally, a smaller consecutive time window (30 ms) for each response condition were employed in this present study, because the modulations of the early ERPs components did not last more than 100 ms.

The software SPSS was used for statistical analysis. The statistical analysis was based on within subject factorial models. The mean amplitude values for each 30 ms time window were entered into repeated measures analysis of variance (ANOVA) with three repeated factors: Laterality (left, midline, right), Frontality (frontal1, frontal2, central, parietal), and Trial Type (congruent vs. incongruent). Significance was set at *p* < 0.05. Degrees of freedom were appropriately adjusted, with the Greenhouse—Geisser (if ε < 0.75) method or Huynh-Feldt method (if 1 > ε >0.75). If one factor with more than two levels has the main effect, Post-hoc analysis was conducted using the Bonferroni test. If the interactions between factors exist, simple effect analysis was conducted to look at the effect of one factor at individual levels of the other factor.

#### 2.4.3 Behavior Data Analysis

Accuracy and mean Response Times (RTs) of every participant were analyzed by SPSS software based on the paired-sample *t* test (two-tail) method.

#### 2.4.4 Topographic distributions Analysis

To study the distribution of the modulations identified by the difference waves (incongruent minus congruent), topographic distributions of the auditory Stroop effects were analyzed by using ERPLAB toolbox. Brain electrical activity mapping for the mean amplitude values of each 30 ms time window at around the seven ERP components (P1, N1, P2, N2, P3, Late-SW1, Late-SW2) were presented.

## Results

### 3.1 Behavior

**Fig 1:**
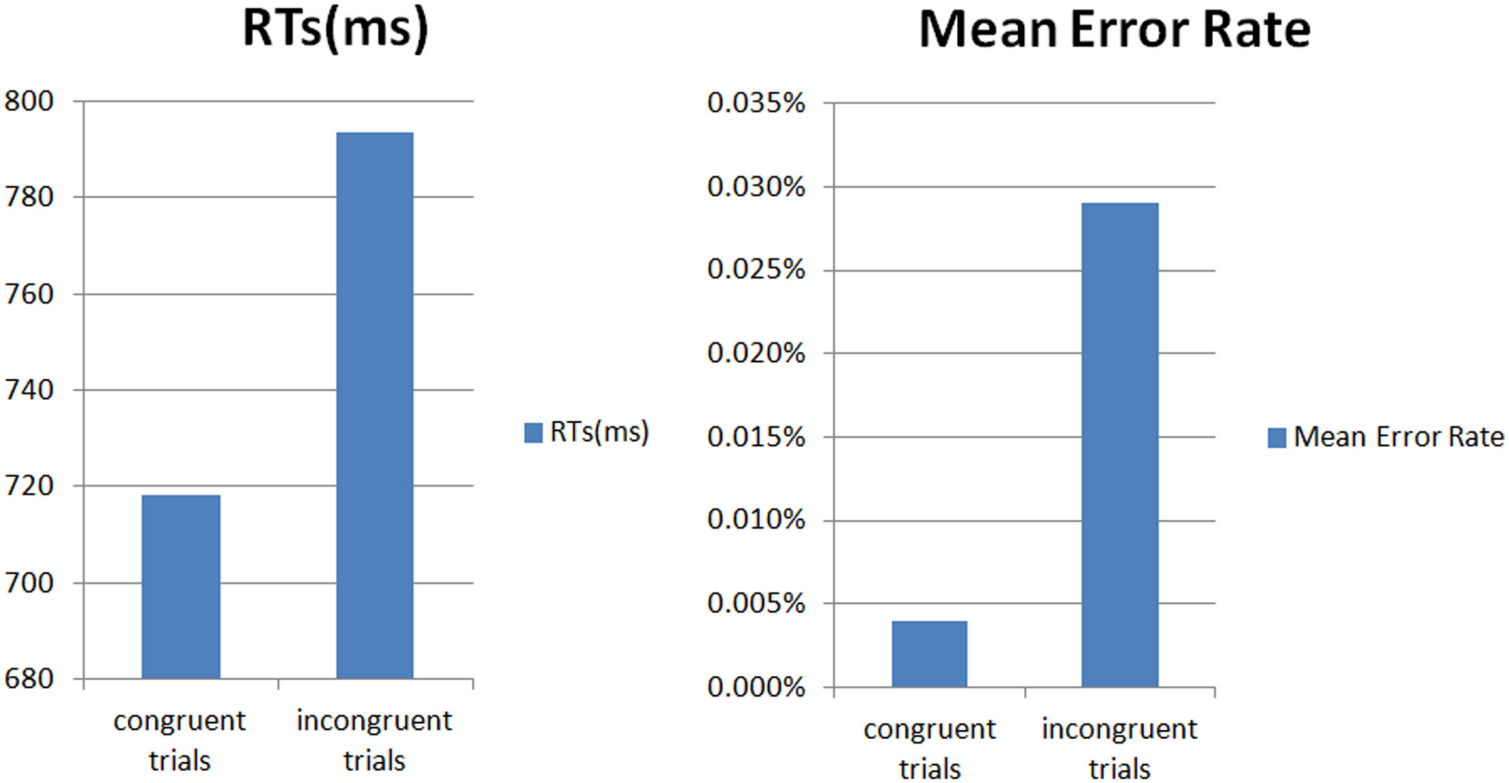
The behavior analysis results.

Fig 1 Shows the behavior analysis results. The accuracy analysis showed that the participants were significantly more accurate for the congruent trials than for the incongruent ones (mean error rates: 0.004% vs. 0.029%, SD = 0.0223, t (20) = 4.9750, p < 0.001). The RTs analysis showed significant slower response for the incongruent trials than the congruent ones (mean RTs: 793.7 ms vs. 718.4 ms, SD = 35.04, t (20) = 9.6123, p < 0.001).

### 3.2 ERPs

#### 3.2.1 ERP components (P1, N1, P2, N2, P3 and Late-SW)

**Fig 2:**
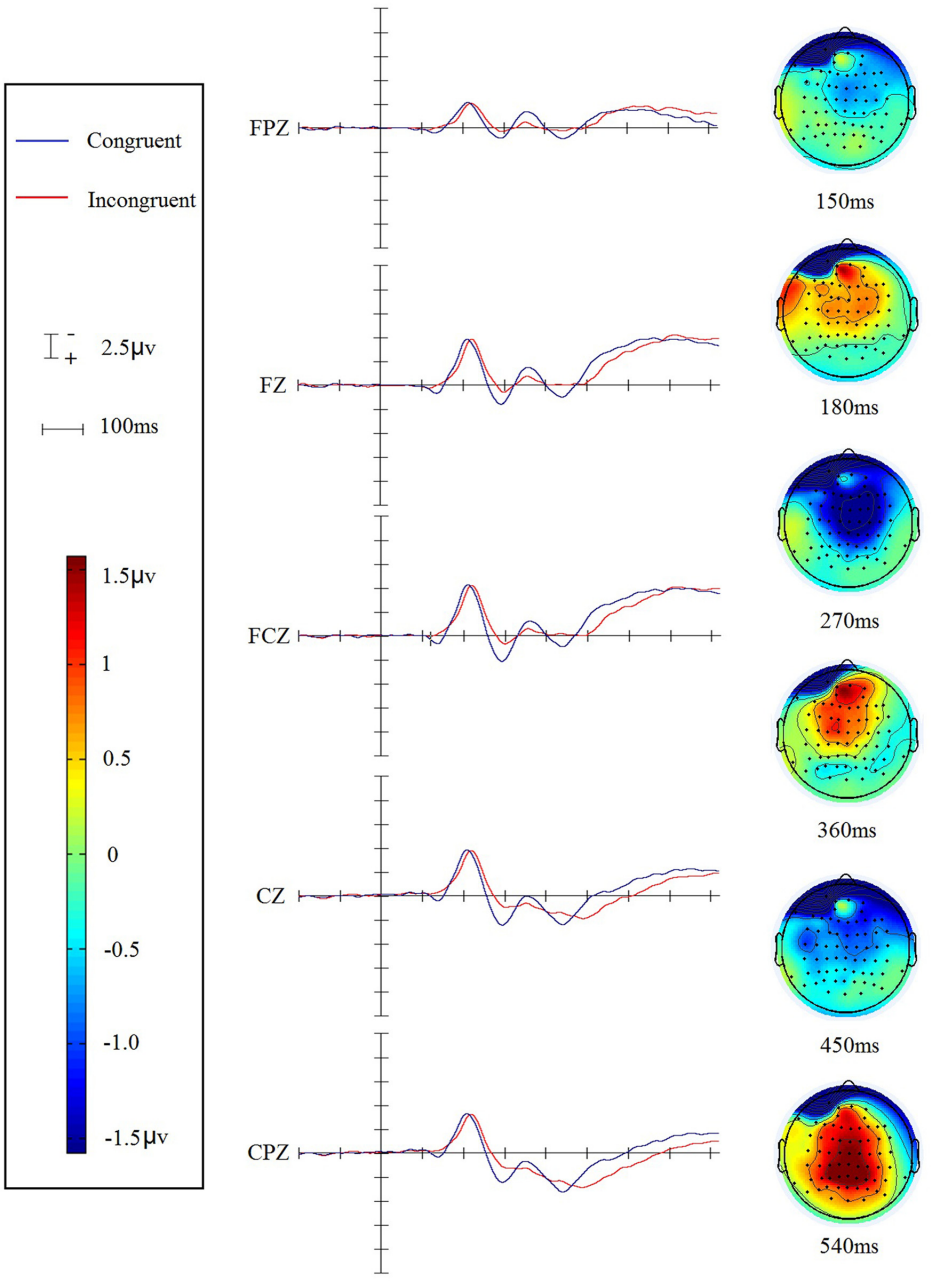
The grand-average ERP waveforms for the five adjacent midline electrodes (elicited by the incongruent stimuli and the congruent ones) and the topological distributions of such ERPs' modulations identified by the group averaged difference waves.

**Fig 3:**
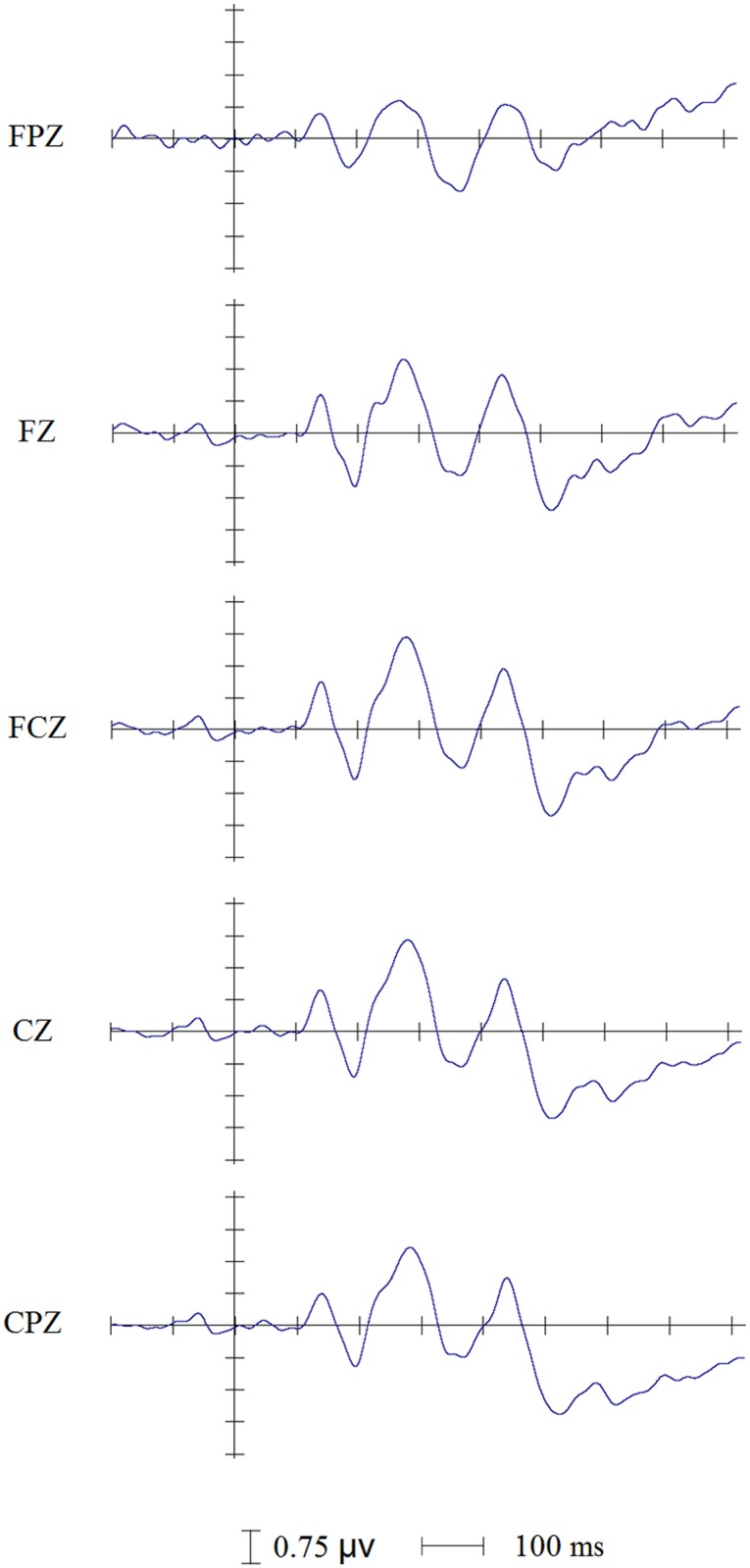
The group averaged difference waves (Incongruent minus Congruent) for the five adjacent midline electrodes showing the four modulations of the P1, the N1, the P2, the N2, the P3 and the Late-SW.

21 participants showed similar waveform morphology in the task including 6 ERP components: the P1, the N1, the P2, the N2, the P3 and the Late-SW (Late slow wave). In this study, all ERP components were delayed about 60 ms than which in other studies, because in this study all stimuli began at around 60 ms but not at 0 ms. These ERP components are presented in Figs 2 and 3. Fig 2 depicts the grand average waveforms elicited by congruent versus incongruent stimuli in the auditory Stroop task and the topographic distributions of such ERPs’ modulations identified by the group averaged difference waves. Fig 3 depicts the group averaged difference waves (incongruent minus congruent) elicited by the auditory Stroop task.

By comparing the amplitude of the ERP waves (between the congruent and the incongruent waves), and inspecting the difference waves (incongruent minus congruent), we identified the main effects similar to (but not completely the same as) the previous auditory or visual Stroop studies in other languages. Similar as the modulation of N2 (the Ninc) peaked at about 300 ms [16], an central distribution declined negativity (the N2, 350~400 ms) between two declined positivity (the P2, peaked around 300 ms; and the P3, peaked around 450 ms) was elicited by the incongruent stimuli in this study. Similar to Henkin’s auditory Stroop study [7], an earlier ERP modulation was found (for the N1, peaked around 200 ms). The N1 of the incongruent trials was more positive than the congruent ones. The Late-SW of the incongruent trials was more negative than the congruent ones in the frontal region while was more positive in the posterior region. Except for the above components, a significant declined P1 (peaked around 130 ms) for incongruent trials were also identified.

The amplitude of these ERP components (P1, N1, P2, N2, P3 and Late-SW) could reflected the cognitive control and conflict processing in there modulations. From the group averaged difference waves (Fig 3, incongruent minus congruent), we could more directly identify these modulations that differentiated the ERPs elicited by incongruent trials from the ERPs elicited by congruent ones. From the Fig 2, we can see that the first modulation reflecting greater positivity for congruent trials peaked at approximate 130 ms; the second modulation reflecting greater negativity for congruent trials peaked at around 200 ms; the third modulation reflecting greater positivity for congruent trials peaked at around 300 ms; the four modulation reflecting greater negativity for the congruent trials beginning at approximate 360 ms; the fifth modulation reflecting greater positivity for congruent trials peaked at around 450 ms. Even though the modulations directions were partly different from the previous findings, these five modulations nonetheless reflected the electrophysiological expressions of pre-execution stage of cognitive control in the auditory Stroop effect. A repeated-measures ANOVA was employed to identify such modulations.

#### 3.2.2 Statistical Analyses

Table 2 shows the main significant results of the repeated-measures ANOVA for the post-stimulus ERPs. Main effects of Trial Type were showed in smaller positive potentials for the incongruent trials relative to the congruent ones (the P1: 121~150 ms; the P2: 241~270 ms, 271~300 ms, 301~330 ms; the P3: 421~450 ms), whereas in smaller negative potentials for incongruent trials relative to the congruent ones (the N1: 181~210 ms; the N2: 331~360 ms, 361~390 ms; the Late-SW1: 481~510 ms, 511~540 ms, 541~570 ms, 571~600 ms, 601~630 ms, 631~660 ms, 661~690 ms; the Late-SW1: 691~720 ms, 721~750 ms, 751~780 ms). In addition, there were significant Trial Type effects with greater negativity for the incongruent trials relative to the congruent ones (the N1: 211~240 ms; the Late-SW2: 781~810 ms).

**Table 2:** Summary of results from ANOVA conducted for post-stimulus ERPs.

Times (ms)	Trial Type	Trial Type *Laterality	Trial Type *Frontality	Trial Type *Laterality *Frontality
**P1**				
121~150	*F* = 5.6, *p* = .029	*F* = 8.3, *p* = .004	NS	NS
**N1**				
181~210	*F* = 10.4, *p* = .004	NS	NS	NS
211~240	*F* = 5.3, *p* = .032	NS	NS	NS
**P2**				
241~270	*F* = 19.4, *p*<0.001	NS	NS	NS
271~300	*F* = 21.3, *p*<0.001	*F* = 4.7, *p* = .021	NS	*F* = 2.7, *p* = .016
301~330	NS	*F* = 3.6, *p* = .042	NS	NS
**N2**				
331~360	*F* = 4.3, *p* = .05	NS	NS	*F* = 2.4, *p* = .032
361~390	NS	NS	NS	*F* = 2.6, *p* = .026
**P3**				
421~450	*F* = 7.4, *p* = .013	NS	NS	NS
**Late-SW1**				
481~510	*F* = 20.4, *p*<0.001	NS	NS	NS
511~540	*F* = 39.6, *p*<0.001	NS	NS	NS
541~570	*F* = 18.8, *p*<0.001	NS	NS	NS
571~600	*F* = 24.8, *p*<0.001	NS	NS	NS
601~630	*F* = 21.3, *p*<0.001	NS	*F* = 6.9, *p* = .004	NS
631~660	*F* = 9.7, *p* = .006	NS	*F* = 8.3, *p* = .003	NS
661~690	*F* = 7.1, *p* = .015	NS	*F* = 14.1, *p*<0.001	NS
**Late-SW2**				
691~720	NS	NS	*F* = 23.7, *p*<0.001	NS
721~750	NS	NS	*F* = 12.0, *p* = .001	NS
751~780	NS	NS	*F* = 16.1, *p*<0.001	NS
781~810	NS	NS	*F* = 14.6, *p*<0.001	NS

*: the interaction between two factors or among three ones;

NS: not significant.

Further simple effect analysis for the interaction effects showed significant main effects for trial type in some region, as showed in below. L*i* reflected the Laterality, *i* = 1, 2, 3 (L1: left; L2: middle; L3: right): A*j* reflected the Frontality, *j* = 1, 2, 3, 4 (A1: frontal1; A2: frontal2; A3: central; A4: parietal)]. Table 3 shows the results of simple effect analyses.

**Table 3:** Summary results of simple effect analyses.

Times (ms)	Trial Type	Trial Type	Trial Type
	*Laterality	*Frontality	*Laterality
			*Frontality
**P1:**			
121~150	L2: *F* = 6.02,*p* = .023		
	L3: *F* = 7.31, *p* = .014		
**P2:**			
271~300	L1: *F* = 18.17, *p*<0.001		L1A1: *F* = 15.46,*p* = .001
	L2: *F* = 20.88, *p*<0.001		L1A2: *F* = 17.41,*p* < .001
	L3: *F* = 23.75, *p*<0.001		L1A3: *F* = 19.25,*p* < .001
			L1A4: *F* = 15.13,*p* = .001
			L2A1: *F* = 14.87,*p* = .001
			L2A2: *F* = 20.49,*p* < .001
			L2A3: *F* = 22.13,*p* < .001
			L2A4: *F* = 20.93,*p* < .001
			L3A1: *F* = 17.93,*p* < .001
			L3A2: *F* = 27.95,*p* < .001
			L3A3: *F* = 26.32,*p* < .001
			L3A4: *F* = 18.50,*p* < .001
301~330	NS		NS
**N2:**			
331~360			L1A1: *F* = 6.38, *p* = .020
			L1A2: *F* = 6.06, *p* = .023
			L1A3: *F* = 5.55, *p* = .029
			L2A1: *F* = 5.40, *p* = .031
			L3A1: *F* = 4.84, *p* = .040
361~390			L1A1: *F* = 6.31, *p* = .021
			L1A2: *F* = 6.21, *p* = .022
			L2A1: *F* = 6.05, *p* = .023
			L1A3: *F* = 5.13, *p* = .035
**Late-SW1:**			
601~630		A1: *F* = 9.60, *p* = .006	
		A2: *F* = 11.37, *p* = .003	
		A3: *F* = 21.76, *p*<0.001	
		A4: *F* = 44.97, *p*<0.001	
631~660		A3: *F* = 10.25, *p* = .004	
		A4: *F* = 26.56, *p*<0.001	
661~690		A3: *F* = 7.67, *p* = .012	
		A4: *F* = 25.87, *p*<0.001	
**Late-SW2:**			
691~720		A4: *F* = 19.45, *p*<0.001	
721~750		A4: *F* = 12.50, *p* = .002	
751~780		A4: *F* = 8.97, *p* = .007	
781~810		A4: *F* = 4.80, *p* = .041	

*: the interaction between two factors or among three ones;

NS: not significant.

Pre-execution Components:

P1 (121~150 ms): The successive analysis indicated that the difference between Trial Type was significant for the middle (L2: *F* = 6.02, *p* = .023) and right (L3: *F* = 7.31, *p* = .014) brain region, with a smaller positivity for the incongruent trials relative to the congruent ones.
P2 (241~330 ms): Simple effect analysis applied to the interactions for the P2 showed that the difference between Trial Type was more significant for right frontal-central area than for other areas [271~300 ms (L1: *F* = 18.17, *p*<0.001; L2: *F* = 20.88, *p*<0.001; L3: *F* = 23.75, *p*<0.001); L1A1: *F* = 15.46, *p* = .001; L1A2: *F* = 17.41, *p*<0.001; L1A3: *F* = 19.25, *p*<0.001; L1A4: *F* = 15.13, *p* = .001; L2A1: *F* = 14.87, *p* = .001; L2A2: *F* = 20.49, *p*<0.001; L2A3: *F* = 22.13, *p*<0.001; L2A4: *F* = 20.93, *p*<0.001; L3A1: *F* = 17.93, *p*<0.001; L3A2: *F* = 27.95, *p*<0.001; L3A3: *F* = 26.32, *p*<0.001; L3A4: *F* = 18.50, *p*<0.001]
N2 (331~390 ms): Significant simple effects were mainly found in the frontal region [331~360 ms (L1A1: *F* = 6.38, *p* = .020; L1A2: *F* = 6.06, *p* = .023; L2A1: *F* = 5.40, *p* = .031; L3A1: *F* = 4.84, *p* = .040); 361~390 ms (L1A1: *F* = 6.31, *p* = .021; L1A2: *F* = 6.21, *p* = .022; L2A1: *F* = 6.05, *p* = .023)], and the center region [331~360 ms (L1A3: *F* = 5.55, *p* = .029); 361~390 ms (L1A3: *F* = 5.13, *p* = .035)] with a smaller negativity for the incongruent trials relative to the congruent ones.

Execution Components:

Late-SW1 (601~690 ms): Significant simple effects were mainly found in the central and the posterior areas [601~630 ms (A3: *F* = 21.76, *p*<0.001; A4: *F* = 44.97, *p*<0.001); 630~660 ms (A3: *F* = 10.25, *p* = .004; A4: *F* = 26.56, *p*<0.001); 661~690 ms (A3: *F* = 7.67, *p* = .012; A4: *F* = 25.87, *p*<0.001)] and the frontal areas [601~630 ms (A1: *F* = 9.60, *p* = .006; A2: *F* = 11.37, *p* = .003)] with a smaller negativity for the incongruent trials relative to the congruent ones.
Late-SW2 (691~810 ms): Significant simple effects were mainly found in the posterior region [691~720 ms (A4: *F* = 19.45, *p*<0.001); 721~750 ms (A4: *F* = 12.50, *p* = .002); 751~780 ms (A4: *F* = 8.97, *p* = .007); 781~810 ms (A4: *F* = 4.80, *p* = .041)] with a smaller negativity for the incongruent trials relative to the congruent ones.

As can be seen in the above results, three-way repeated measures analyses for each 30 ms time window revealed a significant main effect of trial types in all identified ERP components. Almost all the significant main effect of the pre-execution ERP components showed reduced amplitude for the incongruent trials than for the congruent ones. Only the part of the Late-SW2 and the N1 showed enhanced amplitude for the incongruent trials than for the congruent ones. These effects reflected the different brain electrical activities between the incongruent conditions and the congruent ones. Even though they did not continue from the beginning of the information processing to the end of responses, they had reflected the time course of the cognitive control by their different brain distribution.

#### 3.2.3 Brain Electrical Activity Mapping

**Fig 4:**
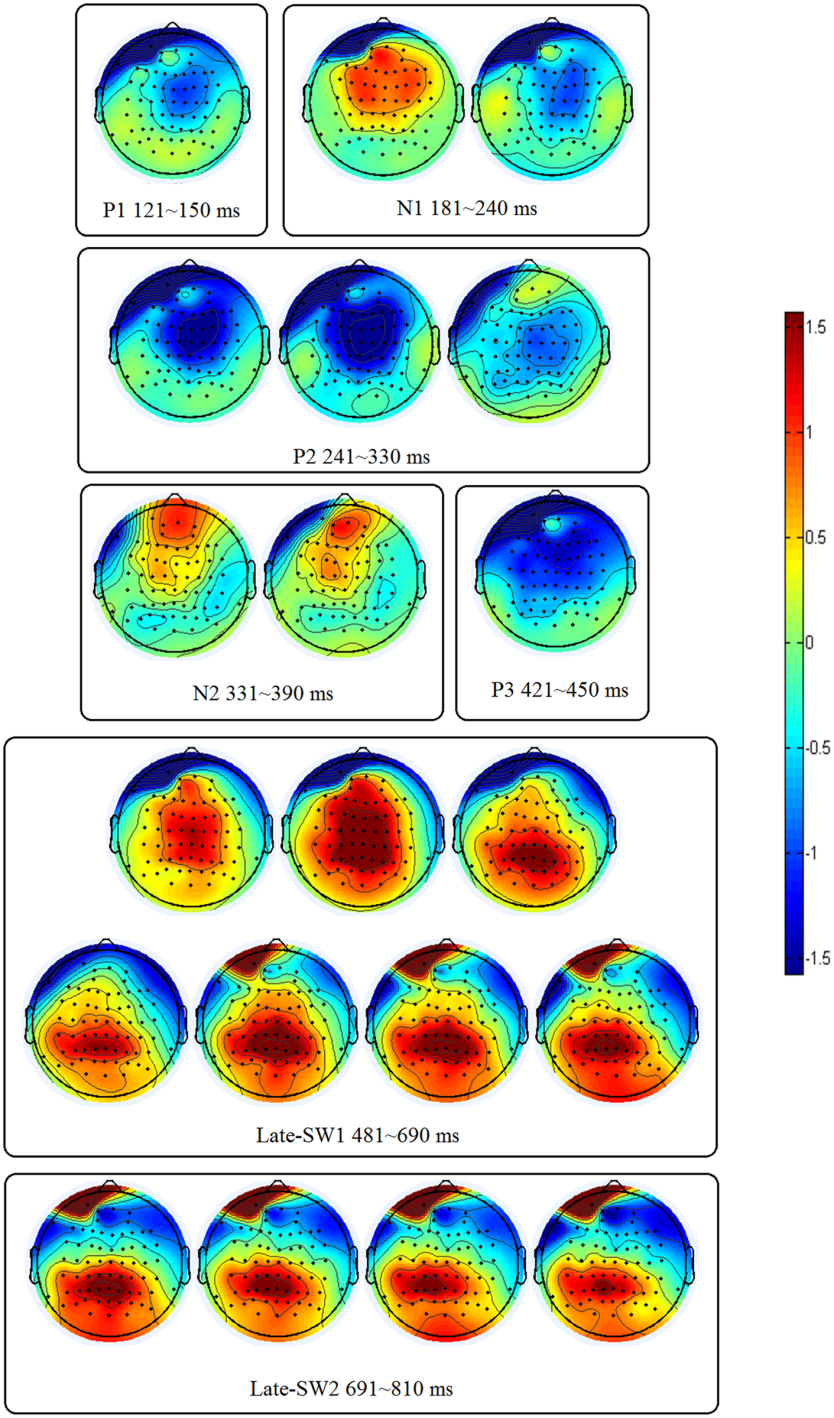
Brain electrical activity mapping (incongruent minus congruent) for the mean amplitude values of each 30 ms time window at around the seven ERP components (P1, N1, P2, N2, P3, Late-SW1, Late-SW2).

Topographic distribution for the difference waves between the incongruent conditions and congruent ones are presented in Fig 4. All modulations of the pre-execution ERP components (P1, N1, P2, N2, P3) were distributed over the frontal and the central region, while the Late-SW2 modulation was distributed over the post-central and the parietal region.

## Discussion

### 4.1 Behavior

The present study used the standard auditory Stroop paradigms like many previous auditory Stroop studies and visual Stroop studies [54]. The results indicated that the Stroop effect were well reflected in the tasks, and showed that there were significantly slower and less accurate responses for incongruent stimuli versus congruent ones. The behavioral data of the present study showed good correspondence with previous auditory or visual Stroop effect studies which using other languages.

### 4.2 ERPs

The aim of the present study focused on exploring the details of the pre-execution ERP components in the auditory Stroop effect, and then confirmed the cognitive control and conflict monitoring mechanism in this study corresponded with which in the previous studies.

The event-related brain activity of this present study revealed seven ERP components (P1, N1, P2, N2, P3, Late-SW1, Late-SW2). The modulations derived from these components showed different details between incongruent and congruent stimuli, and reflected both a pre-execution conflict effect and a response conflict effect. These results were generally compatible to previous auditory studies [16, 45] and visual studies [34, 35], suggesting that the auditory cognitive control processing mechanism might be similar to the visual one on some levels. That is, the general conflict detection-resolution mechanism in cognitive control is supramodal. Moreover, because our oriental languages auditory Stroop effect is similar to the other language versions, it may be concluded that cognitive control has the same mechanism under different language.

In spite of the response conflict effects, we aimed to precisely explore the pre-execution conflict effects, which showed more inconsistency in the previous studies. In those previous auditory studies, Donohue’s work [16] and Buzzell’s work [46] found that there was only an early effect (the Ninc, at about 300 ms, around the N2) involved in conflict detection, and they did not identify the modulations of other pre-execution ERP components. However, the other two studies [7, 45] revealed an auditory modality-specific conflict-processing “signal”, suggesting that the N1 is a mixed component that involves in the conflict detection. Particularly, the present study revealed significant effects both for the N1 and the Ninc. These results suggested that there is a more complex pre-execution processing in cognitive control, and was reflected, at least, in both the N1 and the Ninc.

#### 4.2.1 Pre-execution Components’ Effects

Perceptual Components’ Modulations: the P1, the N1 & the P2 effects.

Similar to the previous auditory Stroop studies [16], the first positive potential in the present study was the P1. In this study, the P1 peaked at the frontal region, and the P1 modulation peaked at the midfrontal area. Those previous auditory Stroop studies [7, 16] did not measure or analyzed whether the P1 modulation was significantly affected by stimulus types. In this study, we found the P1 was significantly declined by incongruent stimulus. Although P1 is considered an obligatory cortical AEPs (auditory evoked potentials) component of the cortical [55], it has small amplitude and latency and P1 modulation was hard to be detected in many experiments. However, our results actually provide a possibility to the points that P1 or other earlier auditory evoked potentials can be modulated by cognitive control mechanism. By investigating data from a visual Stroop task, Klimesch et al. assumed that P1, a manifestation of an evoked alpha wave, reflects a top-down process that "gate" the direction of information processing in the brain [56]. Therefore, in the auditory Stroop task, the P1 may reflect a subject’s selection attention triggering a top-down early stimuli processing.

The first negative potential in this study is the N1 of AEPs, which is also an early ERP consciousness awakening component. Auditory N1 has several different subcomponents, and is thought to be sensitive to the attention and the expectation of the sensory stimuli [48, 57]. In the present study, the topography map for difference wave revealed that the N1 modulation had frontal and central distribution. These results provided support to those conclusions in the previous studies [7, 45] that the N1 modulation reveals an early cognitive control stage related to conflict sensory detection.

Our repeated-measures ANOVA for the ERPs components found a significant effect for the P2 (higher amplitude for congruent trials than incongruent ones). This effect was correspondence with Donohue’s study [16], as the Ninc effect they found exactly consisted of the P2 and the P3 modulations (declined amplitude for incongruent trials). The P2 had left laterality and was distributed over the central and frontal region. The topography map of difference wave also revealed the P2 modulation distributed over the frontal and central region. P2 is typically related to cognitive processes, such as working memory [58–60], semantic processing [61–63], etc.

Altogether, because of the cognitive functions of found by the previous studies and its left laterality, the P2 modulation might involve in the complex pre-execution cognitive control stages which are related to initial semantic information processing.

#### Identification Components’ Modulations: N2 & P3 effects

As for the N2 in the present study, the modulation of this component was also distributed over the frontal and central region in the topography map of difference wave. These findings are similar in temporal order and scalp topography to two previous studies [16]. Donohue suggested that this effect might be at least in part a modulation of N2c, which has been linked to conflict monitoring, and error-related detection [16]. In the present study, our data did not have enough error trials to analyze this possible explanation.

Another possibility is that as the distribution of this effect was similar to the N2b component which is largest over the central region [57, 64], this N2 effect would at least partly reflected an underlying categorization processing like N2b. Two ERP components, MMN (N2a) and N2b, usually contribute to forming N2 [50]. N2b is usually related to detection of stimulus changes and phonological categorization [65, 66]. Therefore, the N2 should be regarded as a pre-execution cognitive control process that categorizes the conflict stimuli information.

Based on the above concerns, we suggested that the N2 was an identification or categorization component. Neurally, the N2 modulation might play an important role in conflict recognition or identification during conflict monitoring, after conflict stimuli firstly pass through the sensory cortex. It would reflect the coding of the conflict information for sending the signal to the conflict control brain areas (executive cortex), in which the conflict resolution would be executed and a response decision would be made.

Moreover, the N2 modulation have similar topological distribution as the N1 modulation, step across the frontal high level region and the language processing region, but showed the left laterality. This distribution suggested that the N2 modulation might reflected a cognitive control stage that involved in translate the execution command as psychological language before signaling the right hand to do a right response.

For the P3 in this present study, it was a parietally maximal P3b component. The P3 amplitude was larger for the congruent stimuli than the incongruent ones. More strictly, this P3 might overlap the Late-SW1, and the delayed latency of the P3 cause to the significant results in the statistic analysis for the P3 and the Late-SW1. The P3 latency have been proposed to be influenced by the processes which are related to response selection and execution [67]. This P3 effect was also partly compatible with some P3 studies on ‘resource allocation’ theory [68] that when a participant need more effort to handle the task, the amplitude of the P3 would be larger. In another words, the incongruent stimulus might arouse cognitive control mechanism to inhibit the resource allocation to irrelevant information, thus declined the amplitude of the P3 wave. Furthermore, the P3 modulation was distributed around frontal region and other region except the parietal region. All in all, the P3 modulation might also reflected a pre-execution or pre-motor cognitive control process.

Both the N2 modulation and the P3 modulation were distributed around pre-motor and supplementary motor cortex (peaked at post-central region and pre-motor areas). It suggested that the N2 and the P3 reflected the pre-motor control stage in cognitive control mechanism that implement the function of signaling the execution cortex and resource allocation of motor pre-execution or response selection.

#### 4.2.2 Executive Components’ Modulation: the Late-SW

The late slow wave (Late-SW) in this study had two sub-components: the Late-SW1 (from 500 to 690 ms) and the Late-SW2 (from 600 to 690 ms). Both the Late-SW1 and the Late-SW2 showed the enhanced positivity in the posterior region. However, in the frontal region, the Late-SW1 showed more declined negativity amplitude for the incongruent trials than for the congruent ones, whereas the Late-SW2 had greater negativity amplitude for the incongruent stimulus than for the congruent ones. Interestingly, the Late-SW1 might have some overlapping with the P3 effects, as the prolong latency of the P3 might also cause to a sustained enhanced positivity potential. The modulation of the Late-SW showed more significance in central and posterior areas, and continued through the reaction time. Although in this study we focused attention on the pre-execution effects, this the Late-SW effect obviously reflected a cognitive control stage which is related to execution control and motor control. This Late-SW effect supported other two previous studies [16, 45] that cognitive control can modulate post-perceptual (or response) processes, and represented the conflict resolution stage in the conflict monitoring mechanism.

#### 4.2.3 Analysis strategies and experimental factors in obtaining different results in the pre-execution ERP components or response conflict effect

It is worth noting that the modulations of some pre-execution ERP components (P1, P2, P3), were not identified in the previous auditory studies. Even the N1 effect, The Ninc were not found simultaneously. There were some limitations in previous analysis strategies and experimental design might be the reasons of these omissions.

An important possibility is the limitation of employing an oversize time window (for instance, Donohue used 100 ms time windows) in amplitude-averaging before statistic analyses (Donohue, Liotti et al. 2012), as the pre-execution ERP components usually do not last more than 100 ms. Another possibility might be that some studies [16, 46] found different amplitudes (by comparing the incongruent and congruent conditions) in many pre-execution ERP components, however their aiming to compare the results with previous studies led to a lack of statistic analyses for other components (P1 and N1), or led to imprecise statistic analyses in identifying specific components which related to the interference effect (P2 and P3). Besides, their finding [16] might mix several ERP components’ modulations (P2, N2 and P3) as one effect, and they roughly named this conflict effect as Ninc but did not precisely named it as the specific ERP components’ modulations (i.e. N2 modulation, P2 modulation). Other two studies using different analysis strategies which might avoid the above problems. However, their results were limited in experimental factors as follow.

In addition to the limitation of the previous data analysis strategies mentioned above, incomplete results of the previous studies might partly due to the experimental details, especially due to the stimuli they used. Henkin [7] employed Hebrew words that with the meaning of /father/ or /mother/. However, the contrast between our stimuli and Donohue’s [16] shows that his stimuli need more semantic processing to extract the property from the word (from the speakers but not the words themselves). As mention above, the function of N2 might be the categorization of the words, more semantic process might reduce the significance of the interference (semantic intrude physical dimension). Similarly, the P2 is an early semantic processing potential. Therefore, we suggested that Henkin’s research revealing no significance in the N2 modulation and the P2 modulation is possibly due to the unnecessary semantic processing. Lew’s stimuli [45] were similar as Henkin stimuli thus revealed similar significant interference effect on N1 but not on N2 or P2. Meanwhile, Donohue’s stimuli [16] were similar to ours, different stimuli can be easily recognized by the first letter of the words, but he did not eliminate the gender difference. His stimuli were spoken by one male speaker, and he did not ask the participants to do some additional tasks which can help them to distinguish the high and low pitch. These two experimental factors might be the reason why Donohue’s did not find significant modulations in other pre-execution components. Buzzell’s stimuli also did not eliminate the gender difference. In addition, Buzzell and colleagues had suggested that the reason for the absence of SP effect in their study might be a relative lack of task difficulty in their experiment [46]. This “relative lack of task difficulty” might partly derive from the sensory processing of voice channel might be more automatic than voice pitch, speaker gender and voice volume, which means the conflict between these physical properties and the word meanings need less control. Thus, the sensory effects as well as the post-perceptual effect in their study were weaker than in the others. For the similar reason, other studies might respectively showed an absence of the post-perceptual effect [7] or showed an absence of the sensory effect [16].

Above all, such limitations in analysis strategies and experimental designs may much likely cause to the incomplete and imprecise measurement of all modulations related to the early stage of cognitive control and conflict monitoring. The present study successfully avoided such limitations, and revealed a complete picture of the cognitive control and conflict monitoring mechanism in the auditory Stroop effect.

### 4.3 Model of the Auditory Stroop Task

In combination with the behavioral data (the responses time were about 700 ms) and the results of the previous studies, the ERP results of the present study indicated that there might exist a more complicated cognitive control process which should contain three stages instead of two (as what was identified in previous researches). Firstly, a perceptual stage representing the complex cognitive control of conflict stimuli sensation is reflected in the combination of the P1 modulation, the N1 modulation and the P2 modulation. Secondly, an identification stage represents the cognitive control of pre-motor signaling (categorization or coding of the conflict information) and is reflected in the combination of the N2 modulation and the P3 modulation. Finally, an execution stage finishes the conflict control and make a response decision/command, representing the conflict resolution, and is reflected in the Late-SW modulation. This cognitive control procedure might not only be presented in the auditory Stroop task, but will also be identified from other auditory cognitive-control-related tasks.

**Fig 5:**
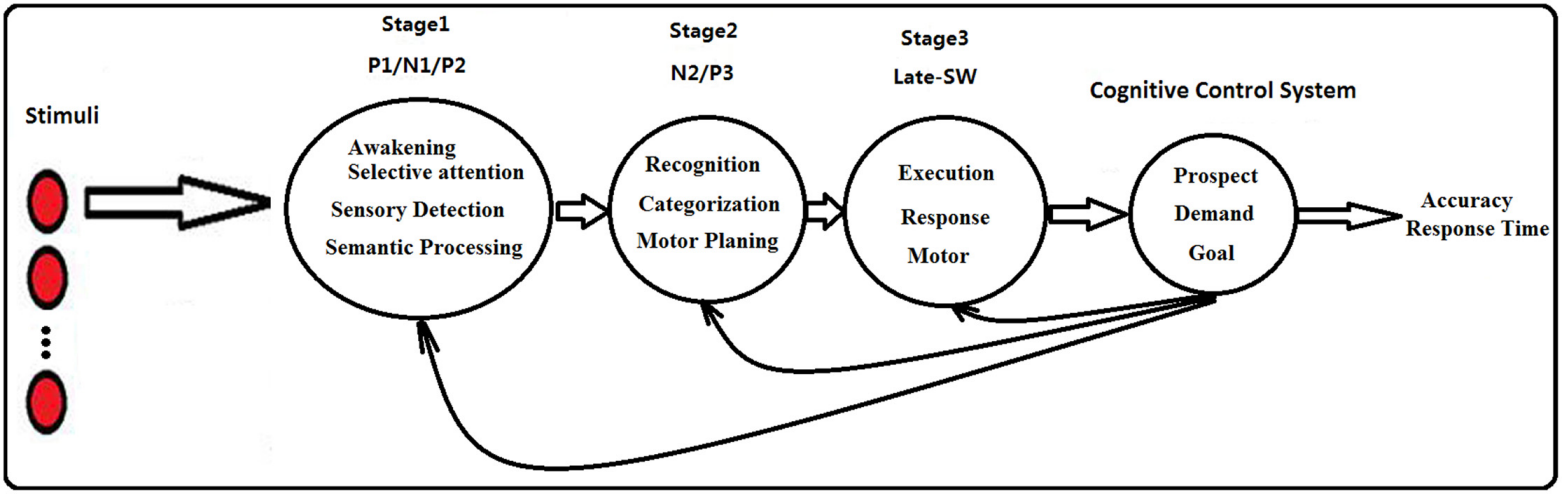
The three stages cognitive control model for auditory Stroop task.

Furthermore, combining with the previous works in the auditory Stroop effect, we characterized a new temporal model—the three stages cognitive control model of the auditory Stroop task. As are showed in Fig 5, the P1 modulation, the N1 modulation and P2 modulation represent the first pre-execution cognitive control stage. The N2 modulation and the P3 modulation is the second pre-execution cognitive control stage. And the Late-SW modulation is the final stage of cognitive control mechanism.

Using this model, we will properly settle the differences of previous researches, as well as the differences between our result and the previous results. All of the ERP waveforms from the previous auditory Stroop effect studies [7, 16, 45, 46] revealed modulations covered three stages of cognitive control, however due to different research emphases (eg. Henkin et al. did not measure the P3 and the Late-SW modulations; Donohue et al and Buzzell et al did not measure the N1 effect and the P1 effect, and they mixed the N2 modulations, the P2 ones and the P3 ones as the Ninc; Lew et al. also did not respectively measure the N2 modulations and the P2 ones) or some other experimental factors (the limitation of their stimuli or statistical time windows, and the lack of task difficulty) mentioned above, they did not successfully identify significant effect for every ERP component. Although inconsistency still remained, these results will not hamper the three stage cognitive control model to be a universally applicable model for the auditory cognitive control processes.

## Conclusion

In the present study, the complete conflict monitoring and cognitive control mechanism especially the pre-execution stage of conflict monitoring, was well observed by using a oriental languages auditory Stroop paradigms.

In the pre-execution stage, we found that the auditory Stroop effect reveals completed ERP modulations (the modulations of P1, N1, P2, N2, P3) for conflict stimuli rather than non-conflict ones. This finding provides evidence for an auditory modality-specific conflict-processing signal and a more detailed conflict monitoring or conflict detecting process in the complex pre-execution stage than which was found in previous studies. In the conflict resolution stage, the Late-SW component in the present study which corresponded to the SP effect in the previous studies was found.

More specifically, we proposed a new cognitive control model, the 3-stage cognitive control model of the auditory Stroop task. The model indicated that a complete cognitive control process includes perceptual detection, identification detection, and conflict resolution during the auditory Stroop task.

In general, the present study using the Chinese language had successfully provided evidence for the conflict monitoring theory and complemented the previous auditory Stroop effect studies in other languages (English and Hebrew language). However, the reduction of the strength of the “signal” (modulations of the pre-execution components) or the absence of the conflict resolution stage would be the results of the limitations in analysis strategies and experimental designs. Therefore, the exploration of the conflict monitoring and cognitive control mechanism in the auditory Stroop paradigms should be carefully designed in future. Moreover, as ERP technique doesn’t fully represent dynamics of EEG data, our further research is studying brain oscillations mechanisms of auditory cognitive control processing.

## References

[1] Egner T, Hirsch J. Cognitive control mechanisms resolve conflict through cortical amplification of task-relevant information. Nat Neurosci. 2005;8(12):1784–90. ISI:000233576200029. 16286928 10.1038/nn1594

[2] Kerns JG, Cohen JD, MacDonald AW, Cho RY, Stenger VA, Carter CS. Anterior Cingulate conflict monitoring and adjustments in control. Science. 2004;303(5660):1023–6. ISI:000188918000049. 14963333 10.1126/science.1089910

[3] MacDonald AW, Cohen JD, Stenger VA, Carter CS. Dissociating the role of the dorsolateral prefrontal and anterior cingulate cortex in cognitive control. Science. 2000;288(5472):1835–8. ISI:000087503800053. 10846167 10.1126/science.288.5472.1835

[4] Swainson R, Cunnington R, Jackson GM, Rorden C, Peters AM, Morris PG, et al Cognitive control mechanisms revealed by ERP and fMRI: evidence from repeated task-switching. J Cogn Neurosci. 2003;15(6):785–99. Epub 2003/09/27. 10.1162/089892903322370717 . 14511532

[5] Botvinick MM, Braver TS, Barch DM, Carter CS, Cohen JD. Conflict monitoring and cognitive control. Psychol Rev. 2001;108(3):624–52. ISI:000170892100006. 11488380 10.1037/0033-295x.108.3.624

[6] Botvinick MM, Cohen JD, Carter CS. Conflict monitoring and anterior cingulate cortex: an update. Trends Cogn Sci. 2004;8(12):539–46. 10.1016/j.tics.2004.10.003 WOS:000202949800006. 15556023

[7] Henkin Y, Yaar-Soffer Y, Gilat S, Muchnik C. Auditory Conflict Processing: Behavioral and Electrophysiologic Manifestations of the Stroop Effect. J Am Acad Audiol. 2010;21(7):474–86. ISI:000281323200006. 10.3766/jaaa.21.7.6 20807483

[8] Most SB. Auditory Stroop reveals implicit gender associations in adults and child and children. 2007.

[9] Cox WM, Fadardi JS, Pothos EM. The addiction-stroop test: Theoretical considerations and procedural recommendations. Psychological Bulletin. 2006;132(3):443–76. ISI:000237839200005. 16719569 10.1037/0033-2909.132.3.443

[10] Stafford T, Gurney KN. Biologically constrained action selection improves cognitive control in a model of the Stroop task. Philosophical transactions of the Royal Society of London Series B, Biological sciences. 2007;362(1485):1671–84. Epub 2007/04/13. 10.1098/rstb.2007.2060 ; Central PMCID: PMC2440779. 17428773 PMC2440779

[11] Herd SA, Banich MT, O'Reilly RC. Neural mechanisms of cognitive control: an integrative model of stroop task performance and FMRI data. J Cogn Neurosci. 2006;18(1):22–32. Epub 2006/01/19. 10.1162/089892906775250012 . 16417680

[12] Soutschek A, Muller HJ, Schubert T. Conflict-specific effects of accessory stimuli on cognitive control in the Stroop task and the Simon task. Experimental psychology. 2013;60(2):140–7. Epub 2012/11/07. 10.1027/1618-3169/a000181 . 23128585

[13] West R. The effects of aging on controlled attention and conflict processing in the Stroop task. J Cogn Neurosci. 2004;16(1):103–13. Epub 2004/03/10. 10.1162/089892904322755593 . 15006040

[14] Qiu J, Luo Y, Wang Q, Zhang F, Zhang Q. Brain mechanism of Stroop interference effect in Chinese characters. Brain Res. 2006;1072(1):186–93. Epub 2006/01/31. 10.1016/j.brainres.2005.12.029 . 16443198

[15] Markela-Lerenc J, Ille N, Kaiser S, Fiedler P, Mundt C, Weisbrod M. Prefrontal-cingulate activation during executive control: which comes first? Cognitive Brain Res. 2004;18(3):278–87. ISI:000220273500006. 10.1016/j.cogbrainres.2003.10.013 14741314

[16] Donohue SE, Liotti M, Perez R, Woldorff MG. Is conflict monitoring supramodal? Spatiotemporal dynamics of cognitive control processes in an auditory Stroop task. Cogn Affect Behav Ne. 2012;12(1):1–15. ISI:000299751400001. 10.3758/s13415-011-0060-z PMC3422637 21964643

[17] Stroop JR. Studies of interference in serial verbal reactions. J Exp Psychol Anim B. 1935;18(6):643–62.

[18] Morgan AL, Brandt JF. An auditory Stroop effect for pitch, loudness, and time. Brain Lang. 1989;36(4):592–603. Epub 1989/05/01. . 2720372 10.1016/0093-934x(89)90088-6

[19] Williams JM, Mathews A, MacLeod C. The emotional Stroop task and psychopathology. Psychol Bull. 1996;120(1):3–24. Epub 1996/07/01. . 8711015 10.1037/0033-2909.120.1.3

[20] Wuhr P. A Stroop effect for spatial orientation. The Journal of general psychology. 2007;134(3):285–94. Epub 2007/09/11. . 17824399 10.3200/GENP.134.3.285-294

[21] Zhao Y, Tang D, Hu L, Zhang L, Hitchman G, Wang L, et al Concurrent working memory task decreases the Stroop interference effect as indexed by the decreased theta oscillations. Neuroscience. 2014;262:92–106. Epub 2014/01/11. 10.1016/j.neuroscience.2013.12.052 . 24406438

[22] Meier ME, Kane MJ. Working memory capacity and Stroop interference: global versus local indices of executive control. Journal of experimental psychology Learning, memory, and cognition. 2013;39(3):748–59. Epub 2012/07/11. 10.1037/a0029200 . 22774858

[23] Soutschek A, Strobach T, Schubert T. Working memory demands modulate cognitive control in the Stroop paradigm. Psychol Res. 2013;77(3):333–47. Epub 2012/03/07. 10.1007/s00426-012-0429-9 . 22391935

[24] Garland EL, Carter K, Ropes K, Howard MO. Thought suppression, impaired regulation of urges, and Addiction-Stroop predict affect-modulated cue-reactivity among alcohol dependent adults. Biol Psychol. 2012;89(1):87–93. ISI:000299714500011. 10.1016/j.biopsycho.2011.09.010 21967855 PMC3245812

[25] Cane JE, Sharma D, Albery IP. The addiction Stroop task: examining the fast and slow effects of smoking and marijuana-related cues. J Psychopharmacol. 2009;23(5):510–9. ISI:000266938800004. 10.1177/0269881108091253 18562413

[26] Ben-Haim MS, Mama Y, Icht M, Algom D. Is the emotional Stroop task a special case of mood induction? Evidence from sustained effects of attention under emotion. Atten Percept Psychophys. 2014;76(1):81–97. Epub 2013/09/18. 10.3758/s13414-013-0545-7 . 24043566

[27] Mama Y, Ben-Haim MS, Algom D. When emotion does and does not impair performance: a Garner theory of the emotional Stroop effect. Cogn Emot. 2013;27(4):589–602. Epub 2012/10/03. 10.1080/02699931.2012.726212 . 23025518

[28] Gootjes L, Coppens LC, Zwaan RA, Franken IH, Van Strien JW. Effects of recent word exposure on emotion-word Stroop interference: an ERP study. International journal of psychophysiology: official journal of the International Organization of Psychophysiology. 2011;79(3):356–63. Epub 2010/12/16. 10.1016/j.ijpsycho.2010.12.003 . 21156188

[29] Milham MP, Banich MT, Barad V. Competition for priority in processing increases prefrontal cortex's involvement in top-down control: an event-related fMRI study of the stroop task. Brain Res Cogn Brain Res. 2003;17(2):212–22. Epub 2003/07/26. . 12880892 10.1016/s0926-6410(03)00108-3

[30] Harrison BJ, Shaw M, Yucel M, Purcell R, Brewer WJ, Strother SC, et al Functional connectivity during Stroop task performance. Neuroimage. 2005;24(1):181–91. Epub 2004/12/14. 10.1016/j.neuroimage.2004.08.033 . 15588609

[31] Milham MP, Erickson KI, Banich MT, Kramer AF, Webb A, Wszalek T, et al Attentional control in the aging brain: insights from an fMRI study of the stroop task. Brain Cogn. 2002;49(3):277–96. Epub 2002/07/26. . 12139955 10.1006/brcg.2001.1501

[32] Adleman NE, Menon V, Blasey CM, White CD, Warsofsky IS, Glover GH, et al A developmental fMRI study of the Stroop color-word task. Neuroimage. 2002;16(1):61–75. Epub 2002/04/24. 10.1006/nimg.2001.1046 . 11969318

[33] Egner T, Hirsch J. The neural correlates and functional integration of cognitive control in a Stroop task. Neuroimage. 2005;24(2):539–47. Epub 2005/01/04. 10.1016/j.neuroimage.2004.09.007 . 15627596

[34] Liotti M, Woldorff MG, Perez R, Mayberg HS. An ERP study of the temporal course of the Stroop color-word interference effect. Neuropsychologia. 2000;38(5):701–11. ISI:000085750800017. 10689046 10.1016/s0028-3932(99)00106-2

[35] West R, Alain C. Event-related neural activity associated with the Stroop task. Brain Res Cogn Brain Res. 1999;8(2):157–64. Epub 1999/07/17. . 10407204 10.1016/s0926-6410(99)00017-8

[36] Larson MJ, Kaufman DA, Perlstein WM. Neural time course of conflict adaptation effects on the Stroop task. Neuropsychologia. 2009;47(3):663–70. 10.1016/j.neuropsychologia.2008.11.013 . 19071142

[37] Roelofs A, Hagoort P. Control of language use: cognitive modeling of the hemodynamics of Stroop task performance. Brain Res Cogn Brain Res. 2002;15(1):85–97. Epub 2002/11/16. . 12433384 10.1016/s0926-6410(02)00218-5

[38] Shor RE. An auditory analog of the Stroop Test. The Journal of general psychology. 1975;93(2d Half):281–8. Epub 1975/10/01. . 1194907

[39] Hamers JF, Lambert WE. Bilingual interdependencies in auditory perception. Journal of verbal learning and verbal behavior. 1972;11(3):303–10.

[40] Cohen G, Martin M. Hemisphere differences in an auditory Stroop test. Percept Psychophys. 1975;17(1):79–83.

[41] Most SB, Sorber AV, Cunningham JG. Auditory Stroop reveals implicit gender associations in adults and child and children. Journal of Experimental Social Psychology. 2007;43:287–94.

[42] Jerger S, Martin RC, Pirozzolo FJ. A developmental study of the auditory Stroop effect. Brain Lang. 1988;35(1):86–104. Epub 1988/09/01. . 3179704 10.1016/0093-934x(88)90102-2

[43] McClain L. Stimulus-response compatibility affects auditory Stroop interference. Percept Psychophys. 1983;33(3):266–70. Epub 1983/03/01. . 6866687 10.3758/bf03202864

[44] Roberts KL, Hall DA. Examining a supramodal network for conflict processing: a systematic review and novel functional magnetic resonance imaging data for related visual and auditory stroop tasks. J Cogn Neurosci. 2008;20(6):1063–78. Epub 2008/01/24. 10.1162/jocn.2008.20074 . 18211237

[45] Lew H, Chmiel R, Jerger J, Pomerantz JR, Jerger S. Electrophysiologic indices of Stroop and Garner interference reveal linguistic influences on auditory and visual processing. J Am Acad Audiol. 1997;8(2):104–18. Epub 1997/04/01. . 9101457

[46] Buzzell GA, Roberts DM, Baldwin CL, McDonald CG. An electrophysiological correlate of conflict processing in an auditory spatial Stroop task: The effect of individual differences in navigational style. Int J Psychophysiol. 2013;90(2):265–71. 10.1016/j.ijpsycho.2013.08.008 WOS:000328722600018. 23994425

[47] Martin BA, Boothroyd A. Cortical, auditory, evoked potentials in response to changes of spectrum and amplitude. J Acoust Soc Am. 2000;107(4):2155–61. Epub 2000/05/02. . 10790041 10.1121/1.428556

[48] Naatanen R, Picton T. The N1 wave of the human electric and magnetic response to sound: a review and an analysis of the component structure. Psychophysiology. 1987;24(4):375–425. Epub 1987/07/01. . 3615753 10.1111/j.1469-8986.1987.tb00311.x

[49] Jun C, Haiyan L, Jijia Z. The latest advances of the Stroop effect—Its theory, paradigms, affecting factors. Psychological Science. 2007;30(2):415–8.

[50] Tome D, Barbosa F, Nowak K, Marques-Teixeira J. The development of the N1 and N2 components in auditory oddball paradigms: a systematic review with narrative analysis and suggested normative values. J Neural Transm. 2014 Epub 2014/06/26. 10.1007/s00702-014-1258-3 . 24961573

[51] May PJ, Tiitinen H. Mismatch negativity (MMN), the deviance-elicited auditory deflection, explained. Psychophysiology. 2010;47(1):66–122. Epub 2009/08/19. 10.1111/j.1469-8986.2009.00856.x . 19686538

[52] Duncan CC, Barry RJ, Connolly JF, Fischer C, Michie PT, Naatanen R, et al Event-related potentials in clinical research: guidelines for eliciting, recording, and quantifying mismatch negativity, P300, and N400. Clinical neurophysiology: official journal of the International Federation of Clinical Neurophysiology. 2009;120(11):1883–908. Epub 2009/10/03. 10.1016/j.clinph.2009.07.045 . 19796989

[53] Winkler I. Interpreting the mismatch negativity. J Psychophysiol. 2007;21(3–4):147–63. ISI:000254577300004.

[54] Rebai M, Bernard C, Lannou J. The Stroop's test evokes a negative brain potential, the N400. International Journal of Neuroscience. 1997;91(1–2):85–94. 9394217 10.3109/00207459708986367

[55] Thabet MT, Said NM. Cortical auditory evoked potential (P1): a potential objective indicator for auditory rehabilitation outcome. International journal of pediatric otorhinolaryngology. 2012;76(12):1712–8. Epub 2012/09/04. 10.1016/j.ijporl.2012.08.007 . 22939592

[56] Klimesch W, Hanslmayr S, Sauseng P, Gruber WR, Doppelmayr M. P1 and traveling alpha waves: evidence for evoked oscillations. J Neurophysiol. 2007;97(2):1311–8. Epub 2006/12/15. 10.1152/jn.00876.2006 . 17167063

[57] Luck SJ. An introduction to the event-related potential technique (cognitive neuroscience). 2005.

[58] Finnigan S, O'Connell RG, Cummins TDR, Broughton M, Robertson IH. ERP measures indicate both attention and working memory encoding decrements in aging. Psychophysiology. 2011;48(5):601–11. ISI:000289151500003. 10.1111/j.1469-8986.2010.01128.x 21039584

[59] Lefebvre CD, Marchand Y, Eskes GA, Connolly JF. Assessment of working memory abilities using an event-related brain potential (ERP)-compatible digit span backward task. Clin Neurophysiol. 2005;116(7):1665–80. ISI:000230296800023. 15908268 10.1016/j.clinph.2005.03.015

[60] Wolach I, Pratt H. The mode of short-term memory encoding as indicated by event-related potentials in a memory scanning task with distractions. Clinical neurophysiology: official journal of the International Federation of Clinical Neurophysiology. 2001;112(1):186–97. Epub 2001/01/04. . 11137677 10.1016/s1388-2457(00)00501-0

[61] Federmeier KD, Kutas M. Picture the difference: electrophysiological investigations of picture processing in the two cerebral hemispheres. Neuropsychologia. 2002;40(7):730–47. Epub 2002/03/20. . 11900725 10.1016/s0028-3932(01)00193-2

[62] Peressotti F, Pesciarelli F, Mulatti C, Dell'Acqua R. Event-related potential evidence for two functionally dissociable sources of semantic effects in the attentional blink. Plos One. 2012;7(11):e49099 Epub 2012/11/29. 10.1371/journal.pone.0049099 ; Central PMCID: PMC3506614. 23189139 PMC3506614

[63] Redmann A, Fitzpatrick I, Hellwig F, Indefrey P. The use of conceptual components in language production: an ERP study. Front Psychol. 2014;5:363 Epub 2014/05/09. 10.3389/fpsyg.2014.00363 ; Central PMCID: PMC4010786. 24808878 PMC4010786

[64] Simson R, Vaughan HG Jr, Ritter W. The scalp topography of potentials in auditory and visual discrimination tasks. Electroencephalography and Clinical Neurophysiology. 1977;42(4):528–35. 66136 10.1016/0013-4694(77)90216-4

[65] Naatanen R, Paavilainen P, Rinne T, Alho K. The mismatch negativity (MMN) in basic research of central auditory processing: A review. Clin Neurophysiol. 2007;118(12):2544–90. ISI:000252204900003. 17931964 10.1016/j.clinph.2007.04.026

[66] Amenedo E, Diaz F. Aging-related changes in processing of non-target and target stimuli during an auditory oddball task. Biological psychology. 1998;48(3):235–67. Epub 1998/10/27. . 9788763 10.1016/s0301-0511(98)00040-4

[67] Doucet C, Stelmack RM. The effect of response execution on P3 latency, reaction time, and movement time. Psychophysiology. 1999;36(3):351–63. . 10352559 10.1017/s0048577299980563

[68] Isreal JB, Chesney GL, Wickens CD, Donchin E. P300 and tracking difficulty: evidence for multiple resources in dual-task performance. Psychophysiology. 1980;17(3):259–73. Epub 1980/05/01. . 7384376 10.1111/j.1469-8986.1980.tb00146.x

